# A Rare Case of Systemic Lupus Erythematosus-Associated Pancreatitis Diagnosed in Trinidad and Tobago

**DOI:** 10.7759/cureus.87773

**Published:** 2025-07-12

**Authors:** Neera Parsan, David King, Christopher Seeraj, Riyad Mohammed

**Affiliations:** 1 Internal Medicine, St. James Medical Complex, Port of Spain, TTO; 2 Physical Medicine and Rehabilitation, St. James Medical Complex, Port of Spain, TTO; 3 Internal Medicine, Port of Spain General Hospital, Port of Spain, TTO; 4 Internal Medicine, St. James Medical Complex, Caroni, TTO

**Keywords:** acute pancreatitis, corticosteroid treatment, internal medicine and rheumatology, serum lipase, systemic lupus erythematosus

## Abstract

Systemic lupus erythematosus (SLE) is a chronic autoimmune disease known for its diverse clinical manifestations and multisystem involvement. While gastrointestinal symptoms are relatively common, pancreatitis remains an uncommon and potentially serious complication. We present the case of a 43-year-old female patient with a known history of SLE who was admitted with progressively worsening abdominal pain. Her evaluation revealed elevated lipase levels and radiological findings consistent with acute pancreatitis. She was managed with corticosteroids and supportive care, resulting in a complete recovery. This case highlights the importance of considering pancreatitis in patients with SLE presenting with abdominal pain and emphasizes the potential benefits of timely immunosuppressive treatment.

## Introduction

Systemic lupus erythematosus (SLE) is a complex autoimmune disease characterized by a relapsing-remitting course and highly variable clinical features. Its manifestations are associated with various autoantibodies, leading to immune complex formation, tissue deposition, and other immune-mediated processes [1]. Globally, the incidence of SLE is estimated at 5.14 (ranging from 1.4 to 15.13) per 100,000 person-years, affecting approximately 0.40 million people annually [2]. Notably, 90% of individuals diagnosed with lupus are female [1]. However, there is a paucity of data on the prevalence of SLE in the Caribbean.

Pancreatitis, an inflammatory condition of the pancreatic tissue, can be either acute or chronic in nature [3]. While gastrointestinal manifestations are common in patients with SLE, pancreatitis is a rare complication, occurring in 0.7%-8.2% of cases and carrying a high mortality rate of up to 30% [4]. The clinical presentation of pancreatitis in patients with SLE does not differ significantly from that in the general population [3]. To date, there has been only one documented case of SLE-associated pancreatitis in the Caribbean. That case, reported in Jamaica by Soyibo et al., describes a clinical picture similar to the one presented here, involving a female patient with a known history of SLE who developed acute pancreatitis [5].

## Case presentation

A 43-year-old female presented to the Port of Spain General Hospital Emergency Department with a three-week history of severe left upper quadrant abdominal pain. Her medical history was notable for a recent diagnosis of hypertension and an 11-year history of SLE. During her previous disease activity, the Systemic Lupus Erythematosus Disease Activity Index 2000 (SLEDAI-2K) score was zero (0), indicating controlled disease. The abdominal pain was described as throbbing and intermittent, eventually becoming persistent and unresponsive to analgesics, including paracetamol and hyoscine butylbromide. She also reported an associated fever but denied any urinary or gastrointestinal symptoms. There was no recent history of unusual food intake or travel. She had no history of gallbladder disease, abdominal trauma, alcohol use, or recent changes to her medications or herbal supplements. Her surgical history was unremarkable.

Her regular medications included hydroxychloroquine 200 mg orally twice daily, paracetamol 500 mg as needed, diclofenac 100 mg orally as required up to a maximum of three times per week, omeprazole 20 mg daily, and a tapering dose of prednisolone, with her current dose being 10 mg daily. She had no known drug or food allergies. Family history was negative for autoimmune conditions, including SLE. She was employed as a registered nurse but was unable to work due to her abdominal pain.

On examination, she appeared to be in painful distress. Her vital signs were as follows: blood pressure 137/94 mmHg, heart rate 107 beats per minute, temperature 37.8°C, respiratory rate 22 breaths per minute, and oxygen saturation (SpO₂) of 96% on room air. A qualitative urine analysis revealed 3+ protein with no blood detected.

Abdominal examination showed tenderness on deep palpation in the left upper quadrant, without rebound tenderness or signs of peritoneal irritation. There was no shifting dullness or fluid thrill. Bowel sounds were normal, and there was no renal angle tenderness. Respiratory examination revealed vesicular breath sounds with adequate bilateral air entry and no crepitations or rhonchi. The remainder of the physical examination was unremarkable.

Initial serum laboratory investigation findings are shown in Table 1 (amylase was not available at the time of hospitalization). The patient's arterial blood gas reports are presented in Table 2.

**Table 1:** Summary of laboratory investigations performed at admission

Test	Value	Normal range
Hemoglobin	10.4 g/dL	12.0 - 16.0 g/dL (Female) / 13.5 - 17.5 g/dL (Male)
White cell count	10.4 × 10^9^/L	4.0 - 11.0 × 10⁹/L
Platelet count	360.8 × 10^9^/L	150 - 450 × 10⁹/L
C-reactive protein	38.6 mg/L	<5 mg/L
Erythrocyte sedimentation rate (ESR)	145 mm/hr.	<20 mm/hr (Male) / <30 mm/hr (Female)
Creatinine	0.9mg/dl	0.6 - 1.2 mg/dL
Blood urea nitrogen	9.1mg/dl	7 - 20 mg/dL
Sodium	137 mmol/L	135 - 145 mmol/L
Potassium	4.5mmol/L	3.5 - 5.1 mmol/L
Chloride	103 mmol/L	98 - 107 mmol/L
Magnesium	1.9 mg/dl	1.7 - 2.3 mg/dL
Corrected calcium	9.66mg/dl	8.5 - 10.5 mg/dL
Alanine aminotransferase	18 U/L	7 - 55 U/L
Aspartate aminotransferase	42 U/L	8 - 48 U/L
Alkaline phosphatase	61 U/L	44 - 147 U/L
Gamma-glutamyl transferase	176 U/L	9 - 48 U/L
Total bilirubin	0.47 µmol/	0.1 - 1.2 mg/dL
Indirect bilirubin	0.1mg/dl	0.2 - 0.8 mg/dL
Direct bilirubin	0.15 mg/dl	0.0 - 0.3 mg/dL
Total protein	7.8g/dl	6.0 - 8.3 g/dL
Albumin	2.3 g/dl	3.5 - 5.0 g
Globulin	4.6g/dl	2.3 - 3.5 g/dL
Lipase	115 U/L	0 - 160 U/L
Total cholesterol	138 mg/dl	<200 mg/dL
Triglyceride	120.60mg/dl	<150 mg/dL
Thyroid-stimulating hormone (TSH)	5.41	0.4 - 4.5 mIU/L
Free thyroxine (fT4)	0.89 ng/dL	0.8 - 2.0 ng/dL
C3 complement	103 g/L	90-180 g/L
C4 complement	12 g/L	10-40 g/L

**Table 2:** Arterial blood gas done on room air

Parameters	Values	Reference range
pH	7.41	7.35 - 7.45
Partial pressure of carbon dioxide (pCO2)	34 mmHg	35 - 45 mmHg
Oxygen saturation (SO2)	98%	95 - 100%
Partial pressure of oxygen (pO2)	92 mmHg	80 - 100 mmHg
Bicarbonate (HCO3)	23.8 mmol/L	22 - 26 mmol/L
Base excess	-6.4 mmol/L	-2 to +2 mmol/L

**Figure 1:**
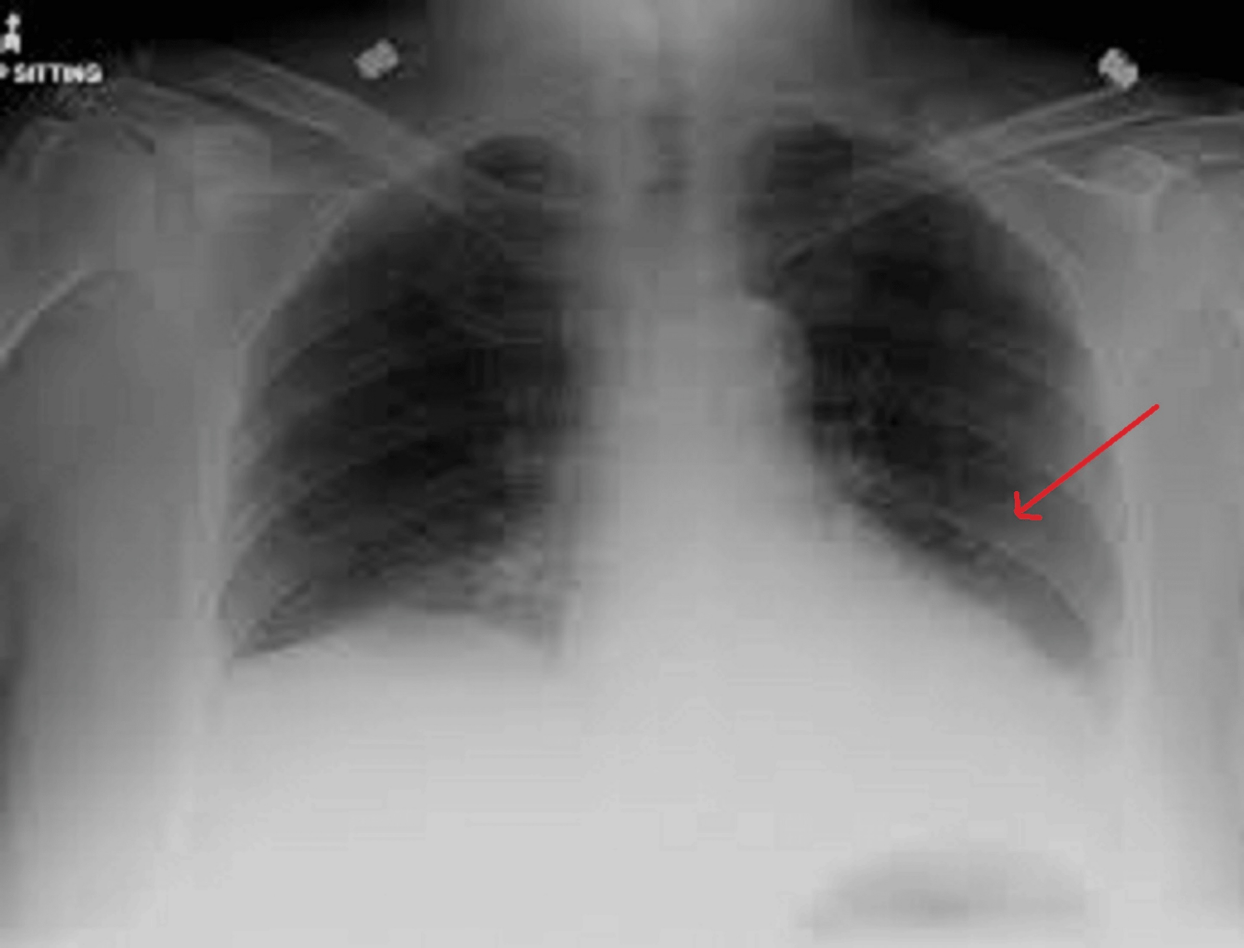
Chest X-ray (posteroanterior (PA) view) showing left lower lobe consolidation. The red arrow indicates the area of consolidation. Source: Radiology department, Port of Spain General Hospital

The chest X-ray revealed a left lower lobe consolidation (Figure 1).

She was diagnosed with community-acquired pneumonia (likely atypical) and suspected SLE-induced acute pancreatitis. She was managed conservatively for pancreatitis with intravenous fluid (0.9% sodium chloride (NaCl)) and tramadol 50 mg IV with antiemetic dimenhydrinate 50 mg IV for analgesia. She was commenced on moxifloxacin 400 mg IV OD to treat the pneumonia for a course of seven days.

**Figure 2:**
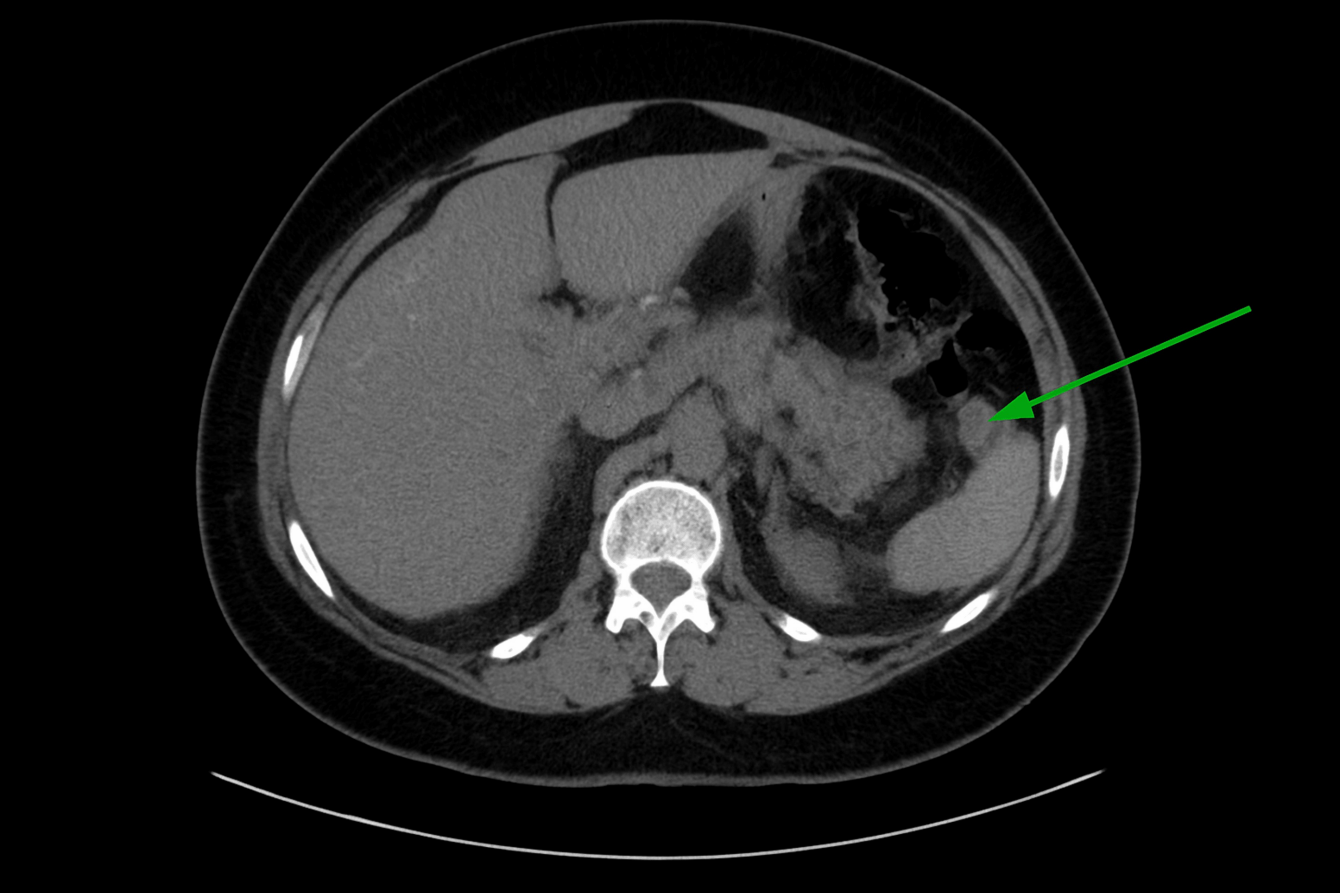
Non-contrast-enhanced computed tomography scan of the abdomen The green arrow indicates the site of fat stranding. Source: Radiology department, Port of Spain General Hospital

On day 2 of hospital admission, a computed tomography scan of the abdomen and pelvis without contrast (Figure 2) was performed, revealing a bulky pancreatic tail with peripancreatic inflammatory changes and adenopathy near the splenic hilum, which was suspicious for pancreatitis or mesenteric panniculitis with a neoplastic change and a small left pleural effusion. There was no evidence of cholelithiasis or biliary duct dilation.

A general surgical consultation was obtained, and she was assessed as having acute pancreatitis secondary to SLE. It was suggested to continue with the current management of intravenous fluid therapy and analgesia.

**Figure 3:**
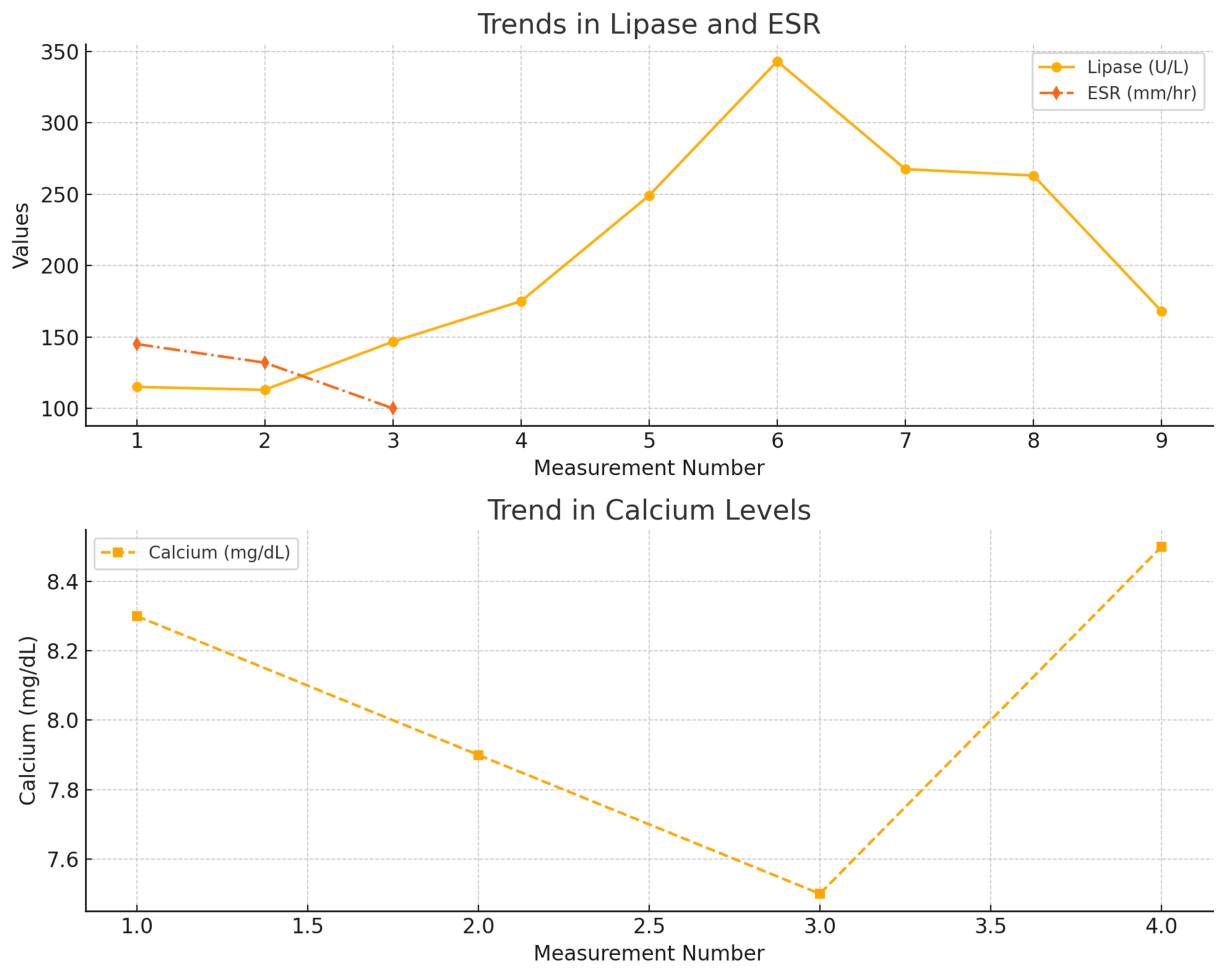
Trend of serum lipase, erythrocyte sedimentation rate (ESR), and serum calcium throughout hospitalization

On the third day of admission, a diagnosis of SLE pancreatitis was confirmed with the rising trend of the lipase from 115 U/L to 249 U/L to 343 U/L (Figure 3). She also complained of persistent abdominal pain, but there had been no clinical deterioration.

Hepatitis B and C serologies and human immunodeficiency virus (HIV) were negative. Blood and urine cultures showed no bacterial growth, and the antibiotics were subsequently discontinued.

**Figure 4:**
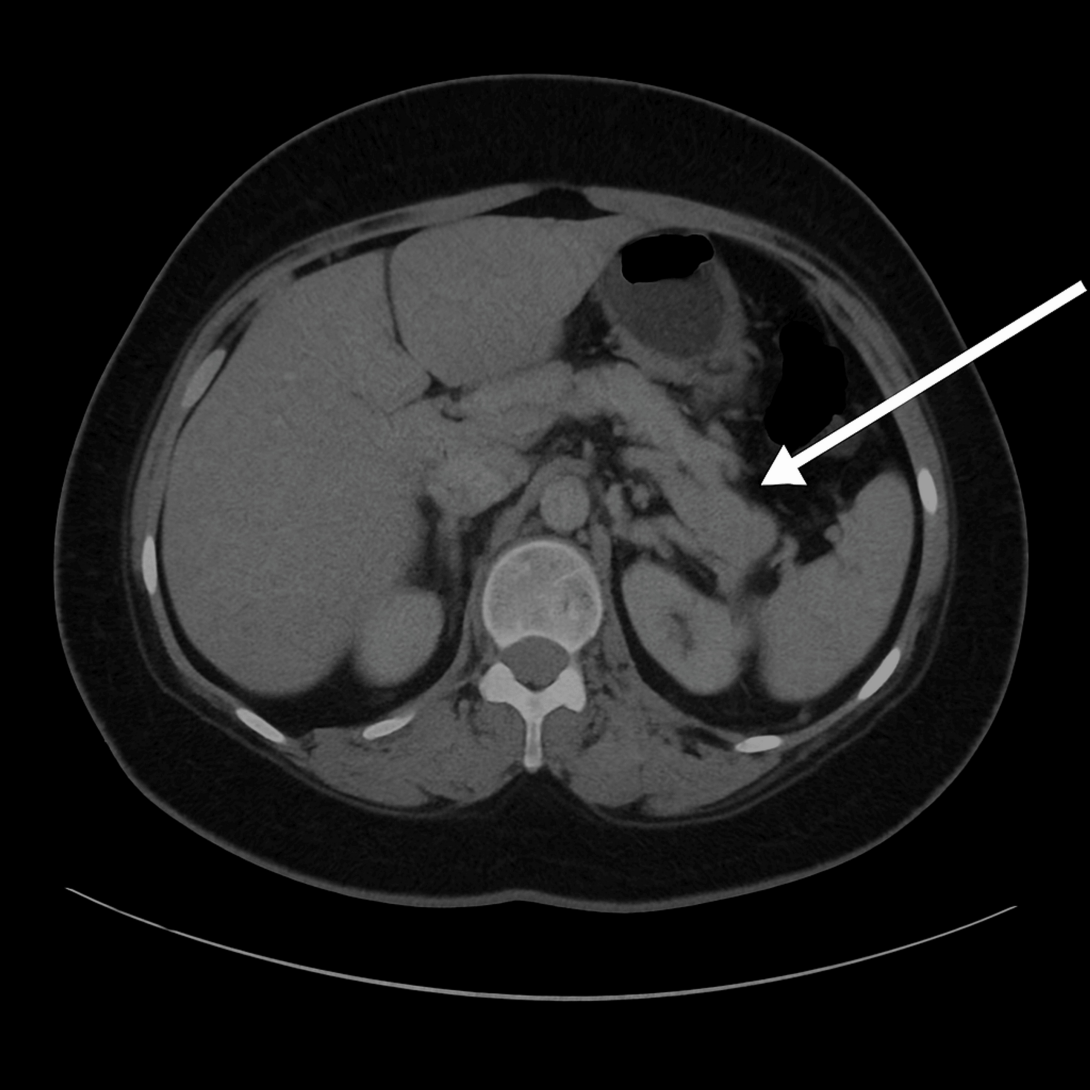
Contrast-enhanced computed tomography scan of the abdomen showing diffuse peripancreatic inflammation. The white arrow highlights the area of fat stranding. Source: Radiology department, Port of Spain General Hospital

On day 6 of admission, a repeat computerized tomography of the abdomen and pelvis with contrast was done (Figure 4) that showed fat stranding with small-volume abdominal free fluid, suggestive of acute interstitial pancreatitis. No inner focal lesions, pancreatic masses, or pancreatic necrosis were seen, and a left pleural effusion that measured up to 5.3 cm.

A rheumatology consultation was obtained, and the patient was started on pulsed methylprednisolone 500 mg intravenously once daily for three days.

Following this treatment, her left upper quadrant abdominal pain significantly improved, and her serum lipase level decreased from 343 U/L to 168 U/L. The patient made a full recovery and was discharged after a 19-day hospitalization. At discharge, her medications included prednisone 60 mg once daily for one week with a weekly taper of 10 mg, amlodipine 10 mg orally once daily, diclofenac 100 mg orally once daily or as needed, and omeprazole 20 mg orally once daily.

At her rheumatology clinic follow-up, she was monitored for recurrence of abdominal pain or other symptoms suggestive of acute pancreatitis, such as nausea, vomiting, fever, or tachycardia, while continuing the prednisolone taper. As the prednisolone dose was tapered to 5 mg daily, hydroxychloroquine 200 mg orally once daily was initiated. At her most recent visit, the patient was stable and doing well on combination disease-modifying antirheumatic drug (DMARD) therapy with azathioprine and hydroxychloroquine.

## Discussion

Acute pancreatitis is an acute inflammatory condition of the pancreas. Acute pancreatitis in adult SLE was first reported by Reifenstein et al. in 1939 as a rare complication in patients presenting with acute abdominal pain [2]. The diagnosis of pancreatitis is primarily based on abdominal pain and elevated serum pancreatic markers, namely lipase and amylase. The management and prognosis vary depending on the severity [6]. It is important to be aware of SLE and its atypical presentations given its rarer complications, such as pancreatitis [7]. Although uncommon, pancreatitis can be associated with viral hepatitis [7]. In this case, hepatitis B and C were negative, and the aspartate aminotransferase (AST) and alanine aminotransferase (ALT) were within normal ranges, making viral hepatitis an unlikely cause. Other common causes of acute pancreatitis must be excluded, such as cholelithiasis, excessive alcohol consumption, corticosteroid use, abdominal trauma, infections such as mumps, malignancy, metabolic disorders such as hypercholesterolemia, hyperparathyroidism, hypercalcemia, previous invasive procedures such as endoscopic retrograde cholangiopancreatography (ERCP), and medications such as azathioprine, loop diuretics, and thiazide diuretics. Although our patient was on corticosteroids, this was ruled out as a cause of the pancreatitis due to the normal triglyceride and calcium levels and the rarity of drug-induced pancreatitis [6, 8-10].

SLE is an autoimmune disorder resulting in multisystem inflammation occurring in 90% more females than men [3, 9, 11]. Common symptoms of gastrointestinal manifestations of SLE are nonspecific, such as nausea, vomiting, diarrhea, anorexia, and abdominal pain. Other common manifestations include oral ulcers, dysphagia secondary to esophageal dysmotility, gastric and peptic ulcer disease, intestinal pseudo-obstruction, inflammatory bowel disease, and pernicious anemia. Pancreatitis in the setting of SLE has an estimated annual incidence of 0.4-1.1 per 1,000 patients [8, 9]. Due to the high number of undiagnosed cases of SLE-related pancreatitis, the frequency of detection is highly underestimated [3, 4].

Most patients present with mild abdominal pain and are often diagnosed as gastroenteritis or peptic ulcer disease [3, 4]. SLE pancreatitis is seen in 0.7%-8.2% of SLE patients [6]. For SLE patients who present with acute abdominal pain, pancreatitis should be a consideration in the differential diagnosis. 

In SLE-related pancreatitis, several pathogenic mechanisms have been described, such as anti-pancreatic antibodies, organ inflammation due to T-cell infiltration, immunocomplex deposition, and complement activation leading to organ ischemia [4]. The normal complement levels of our patient reflect that she was not in an active lupus flare and that this could have been a manifestation of SLE due to a chronic inflammatory state [4, 9]. There is no conclusive evidence to suggest a direct link between complement levels and disease activity in acute pancreatitis [12-14].

A multidisciplinary approach with surgical consultation should always be part of the management plan for patients who present with an acute abdomen. In this case, the patient was referred to surgery due to the abnormal findings on the initial imaging of mesenteric panniculitis, and in the event of the development of complications. In this case presented, pancreatitis was diagnosed based on clinical symptoms and pancreatic enzyme elevation and was supported by characteristic imaging findings. However, SLE patients can have elevation of pancreatic enzymes in the absence of clinical symptoms such as abdominal pain (92%). In asymptomatic SLE patients, lipase is most commonly elevated (50%-70%), followed by amylase (40%-60%). Other enzymes like AST, ALT, and lactate dehydrogenase (LDH) may be mildly elevated in 20%-40% of cases, often due to liver involvement [13-15]. However, the presence of severe abdominal pain together with an uptrending lipase level was sufficient to diagnose pancreatitis in this case. It is important to note that this uptrend in enzymes occurred before empirical steroids were initiated.

Other manifestations of pancreatitis include nausea/vomiting (74%), anorexia, fever (77%), hypoalbuminemia (78%), abnormal transaminases (65%), elevated serum creatinine (44%), and hypocalcemia (23%) [4, 8, 10]. 

Computerized tomographic findings have a diagnostic accuracy of 70% to 90% in cases of acute pancreatitis, regardless of severity, and often require clinicians to correlate with the severity of the disease [9]. A contrast-enhanced computerized tomography was ordered for this patient as it enhances the pancreatic parenchyma, which is useful for diagnostic accuracy [10].

In this case, the patient benefited from steroid treatment as evidenced by her clinical and biochemical response: symptom improvement and resolution, as well as a decrease in lipase. Several studies have shown a decreased mortality rate of 20%-25% with early initiation of steroid treatment [3, 11]. Some studies show a 67% reduction in mortality with early use of corticosteroids, with a 20% mortality rate with corticosteroid introduction versus 61% with no corticosteroids [11].

Aggressive fluid resuscitation has been shown to avoid hypovolemia and its associated complications, such as hypotension, pancreatic hypoperfusion, acute tubular necrosis, ischemic pain, and lactic acidosis [4, 8, 9]. In this case, the patient was hemodynamically stable, and pancreatic enzymes were not elevated enough to indicate pancreatic ischemia. The patient's pain was managed primarily with tramadol 100 mg IV three times a day (TDS) with dimenhydrinate 50 mg IV TDS, which offered satisfactory pain relief. Other analgesics such as morphine, fentanyl, hydromorphone, and nonsteroidal anti-inflammatory drugs (NSAIDs) can be used in the acute setting. Up to 25% of patients with moderate and severe pancreatitis can develop urinary tract infection, pneumonia, and sepsis [4, 11]. Our patient was treated for a community-acquired pneumonia with an appropriate antibiotic agent guided by a microbiology consult, as blood and urine cultures were pending. Antibiotics are not recommended for routine use in acute pancreatitis. They should be used only for confirmed or suspected infections, especially in infected pancreatic necrosis. Treatment typically lasts 14 or more days, adjusted as needed. The goal is to avoid unnecessary antibiotic use and prevent the development of antibiotic resistance [7, 15].

Additionally, limited studies have observed an association between DMARDs and the risk of acute pancreatitis. In a cohort study, approximately 89% with exposure to current glucocorticoid therapy were associated with a decreased risk of acute pancreatitis. A cohort study done by Hopkins showed a 75% favorable prognosis with the use of glucocorticosteroids, whereas patients on DMARD therapy, such as hydroxychloroquine, did not have a beneficial effect [7]. Another study estimated that DMARD use among patients with rheumatoid arthritis (RA) and SLE treated with biologics was a 75% risk reduction for pancreatitis [1, 11]. Although our patient had a long-standing history of hydroxychloroquine use, there is insufficient evidence to support whether this was a protective factor or a major risk reduction for a more sinister presentation of SLE pancreatitis, as evidenced by mild pancreatic enzyme elevations.

There have been reports in the literature of NSAID-induced pancreatitis. However, in a systematic review conducted in 2010 by Pezzilli et al., diclofenac was associated with a lower risk of post-ERCP pancreatitis and was used as analgesia in the treatment of acute pancreatitis with no clinical worsening. In light of this rarity and no defined incidence of NSAID-induced pancreatitis, while it could have been a consideration as a differential diagnosis, it is highly unlikely to have been a cause of the pancreatitis in this case [16]. 

## Conclusions

This case illustrates a young female who is known to have SLE and had an atypical presentation of a three-week history of abdominal pain with acute worsening; she was subsequently diagnosed as having acute pancreatitis based on lipase elevation and the results of computed tomography scans. Although acute pancreatitis is a rare presentation of SLE, it should be suspected in any patient with abdominal pain. Pancreatic enzymes and imaging should be part of the emergency first contact investigations to guide appropriate management. Initiation of early and prompt treatment with corticosteroids decreases the length of hospital stay and reduces mortality.

## References

[1] Rahman A, Isenberg DA (2008). Systemic lupus erythematosus. N Engl J Med.

[2] Reifenstein EC, Reifenstein GH (1939). A variable symptom complex of undetermined aetiology with fatal termination. Arch Int Med.

[3] Azizi S, Jain A, Gutierrez JO, Kaner FB, Khanna R (2023). Acute pancreatitis secondary to acute undiagnosed lupus: a case report. Int J Case Rep Images.

[4] Derk CT, DeHoratius RJ (2004). Systemic lupus erythematosus and acute pancreatitis: a case series. Clin Rheumatol.

[5] Soyibo AK, Alfred R (2010). A case of lupus-associated pancreatitis in Jamaica. West Indian Med J.

[6] Gonzalez A, Wadhwa V, Salomon F, Kaur J, Castro FJ (2019). Lupus enteritis as the only active manifestation of systemic lupus erythematosus: a case report. World J Clin Cases.

[7] Tenner S, Vege SS, Sheth SG (2024). American College of Gastroenterology guidelines: management of acute pancreatitis. Am J Gastroenterol.

[8] Alharbi S (2022). Gastrointestinal manifestations in patients with systemic lupus erythematosus. Open Access Rheumatol.

[9] Brizi MG, Perillo F, Cannone F, Tuzza L, Manfredi R (2021). The role of imaging in acute pancreatitis. Radiol Med.

[10] Breuer GS, Baer A, Dahan D, Nesher G (2006). Lupus-associated pancreatitis. Autoimmun Rev.

[11] Wang F, Wang NS, Zhao BH, Tang LQ (2005). Acute pancreatitis as an initial symptom of systemic lupus erythematosus: a case report and review of the literature. World J Gastroenterol.

[12] Kobayashi S, Yoshida M, Kitahara T, Abe Y, Tsuchida A, Nojima Y (2007). Autoimmune pancreatitis as the initial presentation of systemic lupus erythematosus. Lupus.

[13] Zhang L, Qiao Z, Feng H, Shen J (2020). The early predictive role of complement C3 and C4 in patients with acute pancreatitis. J Clin Lab Anal.

[14] McArthur KE (1996). Review article: drug-induced pancreatitis. Aliment Pharmacol Ther.

[15] Leppäniemi A, Tolonen M, Tarasconi A (2019). 2019 WSES guidelines for the management of severe acute pancreatitis. World J Emerg Surg.

[16] Pezzilli R, Morselli-Labate AM, Corinaldesi R (2010). NSAIDs and acute pancreatitis: a systematic review. Pharmaceuticals (Basel).

